# ATP synthase modulation leads to an increase of spare respiratory capacity in HPV associated cancers

**DOI:** 10.1038/s41598-020-74311-6

**Published:** 2020-10-15

**Authors:** Matthias Kirschberg, Sandra Heuser, Gian Paolo Marcuzzi, Martin Hufbauer, Jens Michael Seeger, Anamaria Đukić, Vjekoslav Tomaić, Slawomir Majewski, Steffen Wagner, Claus Wittekindt, Nora Würdemann, Jens Peter Klussmann, Alexander Quaas, Hamid Kashkar, Baki Akgül

**Affiliations:** 1 Institute of Virology, University of Cologne, Medical Faculty and University Hospital Cologne, Fürst-Pückler-Str. 56, 50935 Cologne, Germany; 2 Institute for Medical Microbiology, Immunology and Hygiene (IMMIH), CECAD Research Center, University of Cologne, Cologne, Germany; 3 Division of Molecular Medicine, Ruđer Bošković Institute, Zagreb, Croatia; 4 Department of Dermatology and Venereology, Medical University of Warsaw, Warsaw, Poland; 5 Department of Otorhinolaryngology, Head and Neck Surgery, Justus-Liebig University, Giessen, Germany; 6 Department of Otorhinolaryngology, Head and Neck Surgery, Medical Faculty, University of Cologne, Cologne, Germany; 7 Institute of Pathology, University Hospital Cologne, Cologne, Germany

**Keywords:** Human papilloma virus, Head and neck cancer

## Abstract

Mucosal and skin cancers are associated with infections by human papillomaviruses (HPV). The manner how viral oncoproteins hijack the host cell metabolism to meet their own energy demands and how this may contribute to tumorigenesis is poorly understood. We now show that the HPV oncoprotein E7 of HPV8, HPV11 and HPV16 directly interact with the beta subunit of the mitochondrial ATP-synthase (ATP5B), which may therefore represent a conserved feature across different HPV genera. By measuring both glycolytic and mitochondrial activity we observed that the association of E7 with ATP5B was accompanied by reduction of glycolytic activity. Interestingly, there was a drastic increase in spare mitochondrial respiratory capacity in HPV8-E7 and an even more profound increase in HPV16-E7 expressing cells. In addition, we could show that ATP5B levels were unchanged in betaHPV positive skin cancers. However, comparing HPV-positive and HPV-negative oropharyngeal squamous cell carcinomas (OPSCC) we noticed that, while ATP5B expression levels did not correlate with patient overall survival in HPV-negative OPSCC, there was a strong correlation within the HPV16-positive OPSCC patient group. These novel findings provide evidence that HPV targets the host cell energy metabolism important for viral life cycle and HPV-mediated tumorigenesis.

## Introduction

In more recent times reprogramming of the energy metabolism has been identified to be one of the hallmarks of carcinogenesis. Increased mitochondrial activity has frequently been associated with unfavorable clinical outcome in a great variety of human cancers, where tumor progression is commonly characterized by an upregulation of glycolysis and impaired mitochondrial activity^1^. This led to the hypothesis of a metabolic switch in cancer cells, a theory which has further been strengthened over the last decades. However, we now know that mitochondrial oxidative phosphorylation (OXPHOS) is not completely abrogated in cancer cells, and that its use may even fluctuate wildly depending on different stages of carcinogenesis, with cancer cells utilizing different energy sources and metabolic pathways to sustain their increased metabolic demands^2^. Although tumor cells cover their energetic demands largely by involving glycolysis, the functional mitochondrial OXPHOS is fundamental not only by providing additional ATP, but also for the production of metabolites that are essential for tumor cell homeostasis and progression. The respiratory chain complexes I–IV maintain a proton gradient, which is required for the generation of ATP by the ATP synthase (F_1_F_0_-ATPase)^3^.

Human papillomaviruses (HPV) are circular double-stranded epitheliotropic DNA viruses with the ability to infect cells of the cutaneous skin as well as the oral or genital mucosa. Such infections may lead to benign but also malign tumors^4^. HPV of genus betapapillomavirus (betaHPV, e.g. HPV8) are known to be associated with skin cancer development, particularly in Epidermodysplasia verruciformis (EV) patients and organ transplant recipients^5–8^. While infections with low-risk HPV types of genus alphapapillomavirus (alphaHPV, eg. HPV6, HPV11) only lead to benign genital warts, high-risk alphaHPV, such as HPV16, are the cause for the development of squamous cell carcinoma (SCC) of the anogenital region^9^. Furthermore, HPV16 is also known to frequently cause oropharyngeal squamous cell carcinoma (OPSCC). In contrast to HPV-negative OPSCC, whose main risk factors are smoking and/or alcohol consumption, patients with HPV-positive OPSCC are characterized by better locoregional control and a better clinical outcome^10^.

Concerning the oncogenic mechanisms of HPV8, our group previously identified that hyperproliferation and invasion of primary skin keratinocytes is induced by the E7 oncoprotein^11–13^. HPV8-E7-induced invasion of keratinocytes is mainly mediated by an interaction between basal keratinocytes and the extracellular matrix, in particular through the α3β1/fibronectin axis, which we demonstrated in organotypic skin cultures. In the same assays we also identified the E7^L23A^ mutant which was not able to trigger keratinocyte invasion^14^. Several cellular interaction partners of HPV8-E7 have been investigated in previous studies^15,16^. To expand our understanding of how oncogenesis is driven by such interactions, drive oncogenesis, we screened for further E7 interaction partners performing yeast-two-hybrid (Y2H) and Co-IP/Mass-spectrometric (Co-IP/MS) experiments. Here, we identified the mitochondrial protein ATP5B as a potential interaction partner of HPV8-E7. ATP5B is one of the subunits of the mitochondrial ATP synthase, also known as complex V, which is a mitochondrial proton pump^17^. Our results demonstrate that this interaction appears to be crucial for meeting the energy demand required for HPV8-E7-mediated cell proliferation and invasion. Strikingly, the E7 proteins of both HPV11 and HPV16 interact even stronger with ATP5B than HPV8-E7. Lastly, we could show that ATP5B over-expression in HPV16-positive OPSCC correlated with improved patient survival, which was in stark contrast to HPV-negative OPSCC.

## Results

### Identification of ATP5B as a novel HPV8-E7 binding partner

**Figure 1:**
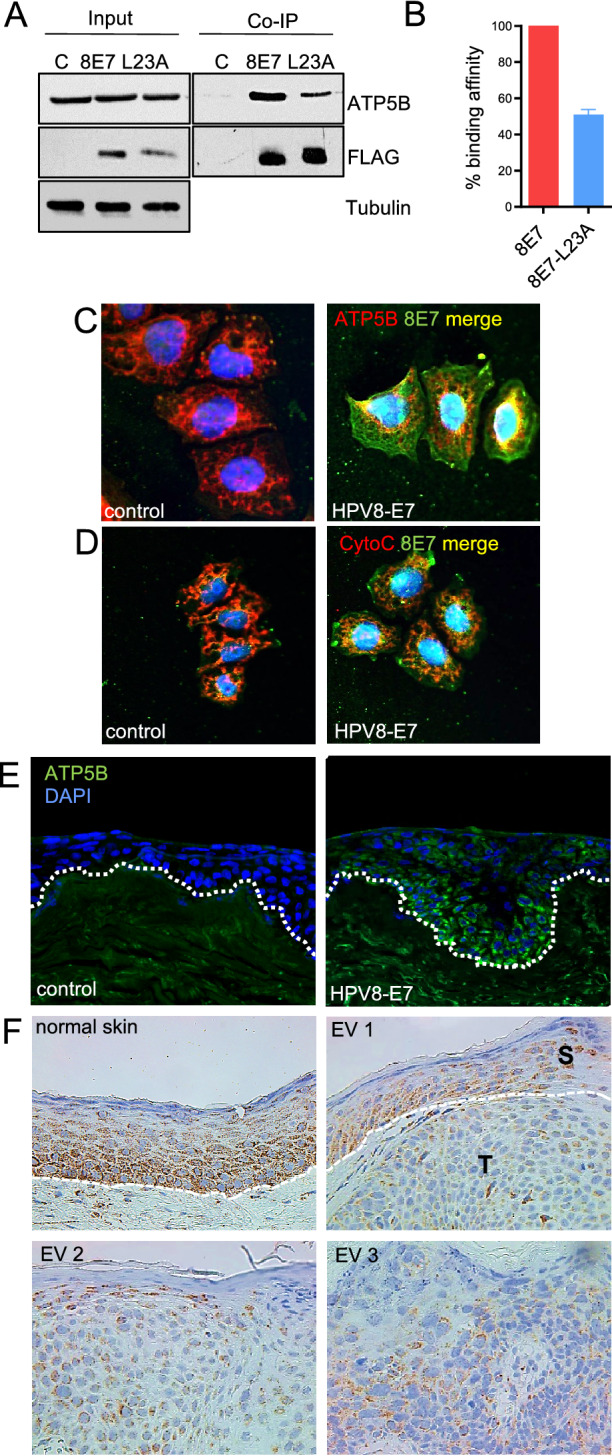
Interaction and co-localization of HPV8-E7 and ATP5B. (**A**) Representative immunoblots of Co-IPs performed with whole protein lysates of C33a cells transiently transfected either with pCMV2-FLAG empty vector, pCMV2-HPV8-E7-FLAG or pCMV-8E7^L23A^-FLAG. Cell extracts were incubated with M2-FLAG agarose beads. Co-immunoprecipitated ATP5B bound to HPV8-E7-FLAG was detected by immunoblotting with specific antibodies. The expression of HPV8-E7-FLAG was confirmed by a Western blot analysis against FLAG. Equal loading was ensured by staining for tubulin as a control. Images were cropped from different gels for better clarity. Original Western blots presented are available in Supplemental Fig. S2. (**B**) Statistical analysis of n = 4 independent Co-IP experiments. (**C**) C33a cells grown on coverslips were immunocytochemically stained to confirm co-localization of HPV8-E7 and ATP5B (DAPI: blue, ATP5B: red, HPV8-E7: green, overlay: yellow, magnification 600 ×). (**D**) C33a cells grown on coverslips were immunocytochemically stained to confirm localization of HPV8-E7 to mitochondria (DAPI: blue, mitochondria-associated protein Cytochrome C: red, HPV8-E7: green, overlay: yellow, magnification 600 ×). (**E**) Immunofluorescence stainings of organotypic skin cultures of N/TERT keratinocytes either harbouring the empty vector pLXSN (left image) or pLXSN-HPV8-E7 (right image). Elevated ATP5B levels are observed in basal and suprabasal cell layers in the HPV8-E7 culture, and lighter signals in more suprabasal areas (magnification 200 ×). (**F**) Representative immunohistochemical staining of normal human skin as well as non-lesional EV skin and EV skin tumors (EV1/EV2 are positive for betaHPV types 5, 36; EV3 is positive for betaHPV types 5, 8, 20, 23, 36, 50) (magnification 400 ×).

To identify novel cellular binding partners of HPV8-E7 we performed both Y2H as well as Co-IP/MS approaches using FLAG-tagged E7 transfected C33a cells. Using a cut-off of more than twofold deregulated and p < 0.05 we ended up with 289 statistically significant targets out of 315 identified proteins via Co-IP/MS (Supplementary Table S1). Of note, we identified 5 subunits of the mitochondrial ATP synthase, ATP5B, ATP5A1, ATP5C1, ATP5F1 and ATP5L as putative binding partners of E7. Interestingly, in Y2H experiments performed in parallel only ATP5B could be confirmed to be interacting with HPV8-E7 (Supplementary Table S2). Therefore, we decided to focus on this novel HPV8-E7 binding partner, which has—to our best knowledge—not been described in previous studies. To further confirm the interaction of ATP5B with HPV8-E7, we performed additional Co-IP experiments with FLAG-tagged HPV8-E7. In the corresponding Western blots we observed co-immunoprecipitation of ATP5B and HPV8-E7 wildtype, with binding of the invasion defective E7 mutant E7^L23A^ to ATP5B being significantly weaker. Total protein levels of ATP5B were not affected (Fig. 1A,B), which was also confirmed by measuring mRNA expression by means of RT-qPCR for cells grown on either uncoated or fibronectin coated tissue culture plates or differentiated in the presence of 2 mM CaCl_2_ (Supplementary Fig. S1). To demonstrate that the interaction between HPV8-E7 and ATP5B occurs at the mitochondria, we performed further immunocytochemical staining for the mitochondrial protein Cytochrome C, ATP5B and FLAG-8E7 as ATP5B can be present in both cytosol as well as the mitochondria. Not only did the staining for ATP5B and E7 showed an overlay (Fig. 1C), but also the staining for Cytochrome C and HPV8-E7 showed a distinct overlay (Fig. 1D), indicating that HPV8-E7 and ATP5B co-localize at the mitochondria.

Despite the observation that total protein levels of ATP5B were not altered in vitro, a moderate upregulation of ATP5B was seen in basal and suprabasal cell layers when HPV8-E7 expressing N/TERT keratinocytes were differentiated as organotypic skin cultures. Diminishing signals were detected in upper layers (Fig. 1E). Lastly, we stained normal human skin and EV skin tissues for ATP5B. In normal human skin as well as non-lesional EV skin ATP5B was expression predominantly detected in the basal and suprabasal epidermal layers. In EV tumor tissues, however, ATP5B was ubiquitously, yet weaker expressed throughout the different tumors (Fig. 1F).

### AlphaHPV types 11 and 16 bind ATP5B stronger than betaHPV type 8, but do not affect its protein expression levels

**Figure 2:**
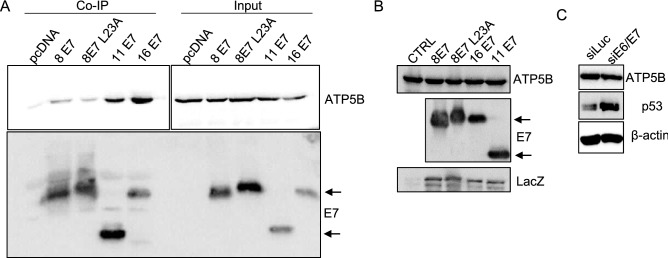
Assessment of ATP5B and E7 binding strengths of HPV11 and HPV16 compared to HPV8-E7. HEK 293 cells were transfected with FLAG-HPV8-E7, FLAG-HPV8-E7L23A, FLAG-HPV11-E7 and FLAG-HA-HPV16-E7. After 24 h cells were harvested and cell extracts were immunoprecipitated with anti-FLAG antibody conjugated beads. Coimmunoprecipitating ATP5B was detected by Western blotting with anti-ATP5B antibody. The protein inputs of ATP5B and E7 are shown. (**B**) HEK 293 cells were transfected with FLAG-HPV8-E7, FLAG-HPV8-E7L23A, FLAG-HPV11-E7 and FLAG-HA-HPV16-E7. After 24 h cells were harvested and residual ATP5B and E7 oncoproteins were detected by Western blot analysis using either anti-ATP5B or anti-Flag antibodies. The expression of β-Galactosidase (LacZ) was used as a control of transfection efficiency. (**C**) HeLa cells were transfected with siRNAs directed against luciferase (siLuc) and HPV18-E6/E7. After 72 h cells were harvested and the levels of ATP5B, p53 (p53 levels were measured to confirm E6E7 knockdown) and the β-actin loading control were detected by Western blotting. Images were cropped from different gels for better clarity. Original Western blots presented are available in Supplemental Fig. S3.

Next, we investigated if ATP5B also forms complexes with E7 of alphaHPV types 11 and 16. HEK293 cells were transfected with FLAG-HPV8-E7, FLAG-HPV8-E7^L23A^, FLAG-HPV11-E7 or FLAG-HA-HPV16-E7 and co-immunoprecipation assays were performed with corresponding cell extracts. The results in Fig. 2A show that endogenous ATP5B co-immunoprecipiated with both alpha and beta HPV E7 proteins, however the strength of interactions between HPV11-E7 and HPV16-E7 with ATP5B were stronger than the interactions of HPV8-E7 and HPV8-E7^L23A^ with ATP5B. Since high-risk alphaHPV E7 oncoproteins have been shown to target some of their substrates for proteasomal degradation^18^, it was of interest to investigate if this was also the case with ATP5B. To this end, HEK293 cells were transfected with FLAG-HPV8-E7, FLAG-HPV8-E7L23A, FLAG-HPV11-E7 and FLAG-HA-HPV16-E7. After 24 h, the cells were harvested and endogenous ATP5B protein levels were analyzed by Western blotting. Interestingly, the results shown in Fig. 2B demonstrate that both high-risk and low-risk alphaHPV E7 oncoproteins did not have any impact on ATP5B protein stability, despite of their strong interactions with ATP5B. To further examine and confirm this observation, we compared ATP5B protein expression levels in cervical tumor derived HPV18-positive HeLa cells in presence and absence of E6/E7. HeLa cells were transfected with siRNA targeting E6/E7 or with luciferase as a control. Cells were harvested after 72 h, and their ATP5B protein levels were determined by Western blotting. P53 protein was also tested and used as a control for E6/E7 ablation. The results shown in Fig. 2C indicate that there are no noteworthy changes in ATP5B protein levels following siRNA treatment against E6/E7. Taken together, these results suggest that ATP5B is not targeted by alphaHPV E7 oncoproteins for proteasomal degradation.

### HPV8-E7 and HPV16-E7 reduce glycolytic activity but increase oxidative respiration

**Figure 3:**
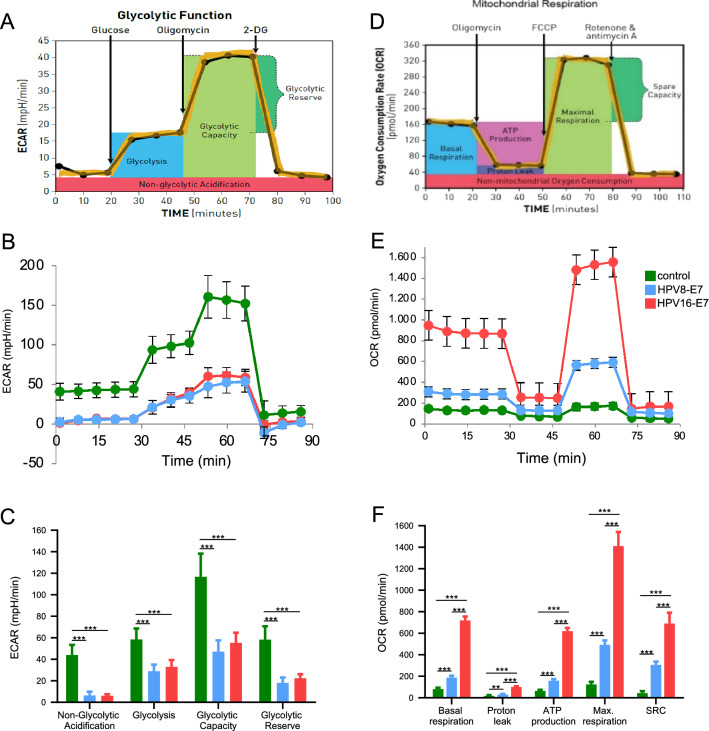
Effect of HPV8-E7 and HPV16-E7 on glycolysis and mitochondrial activity. Schematic principle of the GlycoStress test performed on a Seahorse XF extracellular flux analyzer (**A**) and (**B**) individual parameters for non-glycolytic acidification, glycolysis, glycolytic capacity, glycolytic reserve in N/TERT keratinocytes expressing either the empty retroviral pLXSN, HPV8-E7 or HPV16-E7. Measurements were done in three separate experiments with n = 5 replicates per cell-line. (**C**) Statistical analysis of the individual GlycoStress test parameters (*ECAR* extracellular acidification rate). Schematic principle of the MitoStress test performed on a Seahorse XF extracellular flux analyzer (**D**) and (**E**) individual parameters for basal respiration, proton leak, maximal respiration, spare respiratory capacity, non-mitochondrial respiration and ATP production in N/TERT keratinocytes expressing either the empty retroviral pLXSN, HPV8-E7 or HPV16-E7. Measurements were done in three separate experiments with n = 5 replicates per cell-line. (**F**) Statistical analysis of the individual MitoStress test parameters (*OCR* O_2_ consumption rate, *SRC* spare respiratory capacity).

To characterize the effect of HPV8-E7 and HPV16-E7 binding to ATP5B we next analyzed the effect of these interactions on glycolytic activity of cells by performing the GlycoStress test on the Seahorse analyzer (Fig. 3A). We show that ^RM+^Ntert-8E7, and ^RM+^Ntert-16E7 keratinocytes have a significant reduced glycolytic activity regarding the parameters basal glycolysis, glycolytic capacity and glycolytic reserve (Fig. 3B,C). There was no major difference between the different E7 expressing keratinocytes. To conclude, glycolysis is not being utilized by E7 to meet the heightened energy demands of HPV-E7 positive cells.

We next performed MitoStress tests to measure mitochondrial respiration (Fig. 3D). Regarding basal respiration we observed an about two-fold increase in HPV8-E7 cells in their basal respiration compared to control. This effect was elevated to about tenfold in HPV16-E7 keratinocytes. As far as proton leak is concerned, there was no relevant difference between control and HPV8-E7 cells, but there was a statistically relevant rise in proton leak in HPV16-E7 cells. After addition of FCCP the maximum respiration of control cells remained unaffected, whereas a strong increase in maximum respiration was seen in HPV8-E7 keratinocytes, an effect which appeared even more drastic in HPV16-E7 cells. From our gathered data we calculated an about sixfold increase of the SRC in HPV8-E7 keratinocytes, and an about 14-fold increase in HPV16-E7 keratinocytes. (Fig. 3E,F). E7 drastically upregulates mitochondrial activity which is the key energy source in E7 positive keratinocytes in order to meet their spiking energy demands.

### ATP5B expression in HPV positive and negative OPSCC

**Figure 4:**
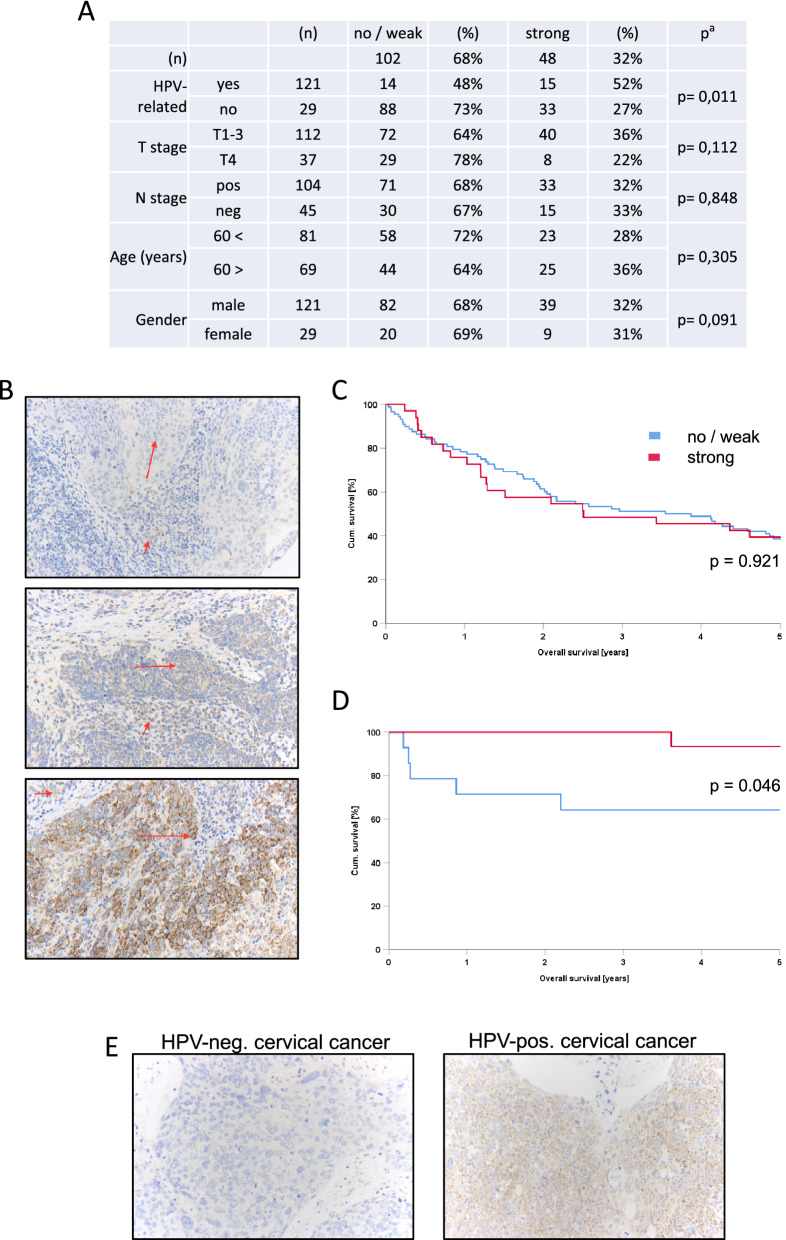
Expression levels of ATP5B in OPSCC and correlation with patient survival. (**A**) Relation of ATP5B expression according to risk factors and tumor/patient characteristics (n = 150). ^a^p values calculated by x^2^ test (Pearson, asymptotic, two-sided), significant p-values (p ≤ 0.05) in bold. (**B**) Shown are three immunohistochemical staining intensities. Negative in carcinoma cells (0), weak expression (2 +) and strong expression in carcinoma cells (3 +). The long arrows mark the carcinoma cells, the short arrows the on-slide control in surrounding inflammatory cells (magnification: 200 ×). Overall survival of patients with HPV-negative (**C**) or positive (**D**) OPSCC correlates with ATP5B expression levels (top: HPV-negative; bottom: HPV-positive). (**E**) Representative immunohistochemical staining of ATP5B son HPV negative (n = 6) and high-risk HPV positive (n = 48) cervical cancer tissues.

To characterize the ATP5B levels in the presence of HPV16 we next examined a tissue micro arrays (TMA) containing in total 207 patient samples originating from both HPV positive and negative OPSCC. The relevant patient characteristics are described in Supplementary Table S3. Out of these 207 samples 150 samples, with 121 HPV negative, and 29 HPV positive OPSCC could be used for the final analysis. We found a significant correlation between positive HPV status and strong ATP5B expression (p = 0.011, Fig. 4A). No such significant correlation was found for T-stage, lymph node metastases, age and gender (Fig. 4A). In line with the observations on OPSCC, we also could detect higher ATP5B levels in high-risk HPV positive cervical cancer tissues (n = 48) compared to HPV negative cancers in which no ATP5B is expressed (n = 6) (Fig. 4E).

In order to determine whether ATP5B expression levels had an effect on overall patient survival of OPSCC patients, we grouped patients with no or weak ATP5B expression (n = 14) and compared them with patients that showed a strong ATP5B expression signals (n = 15) (Fig. 4B). We observed that HPV positive tumors with simultaneously high ATP5B expression have a significantly higher overall survival (p = 0.046) compared to HPV positive patients with no or very low ATP5B expression (Fig. 4C,D). Curiously, no such association was found in HPV negative patients with no / weak (n = 88) or high (n = 33) ATP5B expression (p = 0.921).

## Discussion

Following HPV infection viral oncoproteins drastically alter the host cell machinery to allow for completion of the viral life cycle which is forcing infected cells into proliferation. This may also extend to invasion of keratinocytes, and thus lead to cancer. It remains unknown how particularly the host cell metabolism is controlled by HPV to meet the increased energy demands.

It has previously been described that high-risk alphaHPV early proteins interact with mitochondria^19^. Most recently it has further been demonstrated, that the E6 protein of high-risk alphaHPVs induces the mitochondrial metabolism by increasing the protein levels of various mitochondrial complexes^20^. Here, we now show that E7 of HPV8, HPV11 and HPV16 all interact with varying affinity with the mitochondrial ATP synthase subunit ATP5B, which may therefore represent a conserved interaction across alpha and beta HPV. Total protein levels were, however, not targeted in vitro. The weaker binding of the invasion deficient mutant HPV8-E7^L23A^ to ATP5B allows for the speculation, that residues around L23 may somehow be involved in binding to ATP5B which may be one possible explanation for why the mutant L23A is inferior in driving proliferation and invasion. An explanation for how the E7 proteins tested in our study enter the mitochondria could be that—as predicted by in silico analysis using MitoFates^21^—they all possess a positively charged amphiphilic region near the N-terminus that may aid in facilitating E7 entry into the mitochondria. In conclusion, ATP5B appears to be a conserved mitochondrial target of alphaHPV and betaHPV in the addition previously described nuclear target proteins. Since the low-risk HPV11 E7 protein binds to ATP5B as well suggests that this event might be required for creating a proliferation-promoting environment essential for completion of the viral life cycle as opposed to carcinogenesis per se.

The observation that E7 interacts with ATP5B sparked the idea that it may exert control over mitochondrial energy production. To this end, we studied both glycolytic activity as well as mitochondrial respiration. We observed, as far as glycolysis was concerned, that it was largely abolished in HPV8-E7 and HPV16-E7 positive cells compared to control cells. This is a curious observation as most tumors re-purpose mitochondria and primarily employ glycolysis to generate the energy required for further tumor progression, particularly in hypoxic regions of solid tumors. Strikingly, HPV8-E7 and HPV16-E7 drastically increased the spare respiratory capacity of mitochondria with HPV16-E7 doing so with much greater efficacy than HPV8-E7. Our metabolic data strongly imply, that HPV infected cells have the ability to react to the rising energy requirements. This might be facilitated by boosting ATP synthase activity, causing it to go into overdrive to generate the required energy for either completion of the viral life cycle, benign or malignant keratinocyte transformation.

In addition to the in vitro experiments, we also characterized ATP5B expression levels in vivo in skin tumors of EV patients (betaHPV positive) and in HPV16 positive and HPV negative OPSCC. In line with the comparatively moderate binding of HPV8-E7 to ATP5B, there was only a moderate elevation in total ATP5B levels in stained OPSCC tissues. However, in accordance with the strong binding affinity of HPV16-E7 to ATP5B we found a significant association of high ATP5B expression in HPV16 positive OPSCC. Curiously, high ATP5B levels positively correlated with overall survival of HPV16 positive OPSCC patients, whereas changes in ATP5B expression played no role in HPV negative OPSCC in respect to their overall survival. However, why the mitochondrial metabolism remains so active in HPV positive cells remains unknown. Previous works have shown that HPV negative head and neck squamous cell carcinoma cells seemingly prefer glycolysis rather than OXPHOS. In contrast, HPV positive HNSCC strongly favor OXPHOS for energy generation^22–25^. Cruz-Gregorio et al. recently showed that HPV16 increases protein levels of subunits of mitochondrial complex I–IV as well as the ATP synthase, leading to increased mitochondrial mass. Furthermore, E6 thus increases basal and leak respiration, which was associated with oxidative stress by increasing the amount of reactive oxygen species without having an effect on ATP-linked mitochondrial activity^20^. From these results the authors concluded that this may partially explain why HPV positive OPSCC are more radiosensitive due to oxidative stress and the resulting DNA damage and the susceptibility to ionizing radiation. Our data regarding E7 further support this theory, as we could show that the mere presence of E7 was enough to both increase ATP production at the expense of higher proton leakage, which may also lead to oxidative stress. This would also explain why HPV positive OPSCC patients with high ATP5B expression have a significantly more favorable clinical outcome. The further characterization of these metabolic changes may therefore be invaluable for the identification prognostic markers or novel drug targets.

## Methods

### Yeast-2-hybrid

In order to identify cellular proteins binding to HPV8-E7 a Matchmaker two-hybrid system was used together with a HaCaT cDNA library (Clontech, Saint-Germain-en-Laye, France). Experiments were performed according to the manufacturer`s recommendations.

### Cell culture

N/TERT keratinocytes^26^ were cultivated either in KGM-Gold (containing low (0.05 mM) Calcium) (Lonza, Cologne, Germany) or in RM + medium (consisting of a 3:1 ratio of Dulbecco's modified Eagle's medium [DMEM]-F12 with 10% fetal calf serum [FCS], 1% glutamine, 0.4 μg hydrocortisone, 10^−10^ M cholera toxin, 5 μg/ml transferrin, 2 × 10^−11^ M liothyronine, 5 μg/ml insulin, 10 ng/ml epidermal growth factor, 1 × penicillin–streptomycin mixture)^27^. Retroviral transduction of N/TERT keratinocytes was performed using the retroviral vector pLXSN and the genes encoding for HPV8-E7, HPV8-E7^L23A^ or HPV16-E7 as previously described^14,27^.

Organotypic skin cultures based on de-epidermalised human dermis as matrix and retrovirally transduced keratinocytes were generated as previously described^14^.

For the generation of FLAG tagged HPV8-E7 or HPV8-E7^L23A^, the E7 gene was amplified via PCR and cloned into the pCMV2-FLAG vector via the BamHI site. The L23A mutation in HPV8-E7 was introduced by site-directed mutagenesis^14^. The HPV-negative C33A cell line was cultured in DMEM, supplemented with 10% FCS and 1% Penicillin/Streptomycin. C33A cells were transfected in 6-wells using the CaCl_2_-method with either the empty expression vector pCMV2-FLAG or pCMV2-HPV8-E7-FLAG, pCMV2-HPV8-E7^L23A^-FLAG constructs. HEK-293 cells were transfected in 10 cm^2^ dishes using the CaCl_2_ method either with the empty expression vector pCMV2-FLAG or pCMV2-HPV8-E7-FLAG, pCMV2-HPV8-E7^L23A^-FLAG, Flag-HA-tagged pCMV HPV-16 E7 and FLAG-tagged pCMV HPV-11 E7^28^. siRNA transfections were performed as previously described^29^. In brief, HeLa cells (HPV18 positive) were cultured in Dulbecco’s modified Eagle’s medium (DMEM) with 10% FCS and penicillin/streptomycin. HeLa cells were seeded in 6 cm^2^ dishes and transfected using DharmaFECT transfection reagent (Horizon Discovery, Germany) according to the manufacturer’s instructions with siRNA against luciferase (Horizon Discovery) as the control and siRNA against 18E6/E7 (5′ CAUUUACCAGCCCGACGAG) (custom ordered form Horizon Discovery).

### Co-IP

10 µl of α-FLAG M2 affinity resin (Sigma-Aldrich, Taufenkirchen, Germany, A229) were equilibrated with 0.1 M LSDB buffer (1 mM DTT, 1 mM PMSF) for each sample and centrifuged at 7000 rpm for 2 min at 4 °C. The beads were resuspended in 85 µl 0.1 M LSDB and transferred into a new tube. Then, 300–500 µg of whole protein lysate containing FLAG-fusion proteins were added to the beads, and the mixture was filled up to 1 ml with 0.1 M LSDB, before incubating them on a rotator for 3 h at 4 °C. This step was followed by centrifuging at 7000×*g* for 5 min at 4 °C. Afterwards, supernatants were discarded and samples were washed five times in 1 ml 0.1 M LSDB, before reconstituting them in 30 µl of 0.1 M LSDB. Then, 10 µl of 4 × SDS loading puffer were added and samples were incubated for 5 min at 95 °C. After centrifuging the samples at 7000×*g* for 5 min the supernatants were separated by means of SDS–PAGE and subsequent Western Blots, probing for ATP5B (Sigma Aldrich, HPA001520) or FLAG (Sigma-Aldrich, F4042).

### Western blot

Western blots were performed as described previously^30^. Briefly, cell pellets were resuspended in RIPA buffer containing protease inhibitors, incubated on ice for 30 min, sonicated and then centrifuged at 15,000×*g*, at 4 °C for 15 min. Protein concentrations were measured with the PierceTM BCA Protein Assay Kit (ThermoFisher, Dreieich, Germany). SDS Gels were transferred upon completion to nitrocellulose membranes, which were then blocked with 5% dry milk in TBST. The primary antibodies used targeted ATP5B (HPA001520, Sigma Aldrich), FLAG (F4042, Sigma-Aldrich), anti-p53 (DO-1, Santa Cruz Biotechnology), β-actin (Sigma-Aldrich), β-galactosidase (LacZ, Promega) or tubulin (ab6160, Abcam, Cambridge, UK) and incubated for 2 h at RT or overnight at 4 °C. Following washing, membranes were incubated with corresponding secondary antibodies, after which the membranes were developed using BM Chemiluminescence blotting substrate (Sigma-Aldrich). Western Blots were either visualized using an ECL-hyperfeature film (Amersham, Freiburg, Germany) or by employing the GelDoc system from BioRad (Feldkirchen, Germany).

### Sample preparation for mass-spectrometry

Co-immunoprecipitated cellular proteins bound to HPV8-E7-FLAG were processed and analyzed at the CECAD Proteomics facility, University Hospital Cologne. Afterwards in-solution digest of proteins was performed with reagents provided by the facility: 50 × Protease Inhibitor cocktail (Roche), triethylammoniumbicarbonate (TEAB), 50 mM, urea buffer: 8 M Urea in 50 mM TEAB, benzonase HC nuclease , dithiothreitol (DTT), chloroacetamide, trypsin protease, 1 μg/μL or 0.1 μg/μL, lysyl Endopeptidase (Lys-C), 0.5 μg/μL , formic acid, 10% in water. Firstly, urea lysis buffer was prepared by adding 50 × protease inhibitor to 8 M Urea/50 mM TEAB buffer. Protein lysis was achieved by resolving Urea at 4 °C on a rotator using the concentrated supernatants as solvent to arrive at a final concentration of 8 M. Afterwards samples were centrifuged for 15 min at 20,000×*g* to remove any cell debris. Afterwards protein concentration was measured and 400 µg per sample were transferred to a new 1.5 ml Eppendorf tube. To reduce background noise for the mass spectrometric analysis a sample containing only medium was included. Afterwards DTT was added to each sample at a final concentration of 5 mM, briefly vortexed and then incubated at room temperature for 1 h. Afterwards CAA was subsequently added at a final concentration of 40 mM, the sample vortexed and incubated in the dark for 30 min, before adding Lys-C protease at an enzyme:substrate ratio of 1:75, followed by another incubation period of 4 h at 25 °C. Following this step samples were diluted with 5 mM TEAB to achieve lower urea concentration of 2 M. Then trypsin was added at an enzyme:substrate ratio of 1:75 and the samples were incubated at 25 °C overnight. The following day samples were acidified by adding 1% formic acid to stop the enzymatic reaction. Afterwards, proteins within the supernatants were desalted and further purified using in-house-packed seppack stage-tipps (Waters GmbH, 65760 Eschborn, Germany). The columns were primed with 1 × 1 ml 100% acetonitrile, followed by three washing steps with three times with 3 ml 0.1% formic acid. Afterwards the supernatants were loaded onto stage tip purification columns, which were then washed three times with 3 ml 0.1% formic acid. The stage tips were then transferred to new Eppendorf tubes and proteins were eluted with 300 µl acetonitrile/trifluoroacetic 0.1% acid per sample. Afterwards the eluates were concentrated using a speed vac prior to mass spectrometric analysis.

### Mass-spectrometry

Mass-spectrometric analysis was performed as recently described^31^. For tandem mass spectrometry LC–MS/MS analysis (LC–MS/MS or MS2) and easy nLC 1000 (Thermo Scientific) were coupled to the quadrupole-based Q Exactive Plus (Thermo Scientific) instrument using a nano-spray ionization source. All mass spectrometric proteomics raw data were analyzed using the bioinformatics tools MaxQuant (v1.5.3.8) running with default parameters and Perseus (v1.5.0.3.1). Briefly, MS2 spectra were searched against the Uniprot HUMAN.fasta (downloaded at: 16.6.2017) database, including a list of common contaminants. False discovery rates (FDR) on protein and post-translational modification (PSM) levels were evaluated by a target-decoy approach to 1% (Protein FDR) and 1% (PSM FDR) respectively. The minimal peptide length was set to seven amino acids and carbamido-methylation at cysteine residues was considered as a fixed modification. Oxidation (M) and Acetylation (Protein N-term) were included as variable modifications and match-between runs option was enabled. LFQ quantification was enabled using default settings.

### Seahorse assay for measurement of glycolytic stress

Measurement of cellular glycolytic activity was performed using the Seahorse XF96e analyzer (Agilent Technologies, Massachusetts, USA). N/TERT-HPV8-E7, N/TERT-HPV16-E7 and control cells were grown in RM + medium and seeded out on Seahorse XF96 cell culture microplates 16 h prior to measurement at a cell density of 25,000 cells per well. Assay medium was prepared on the day of the assay by supplementing Seahorse XF Base Medium with 2 mM glutamate and no other additives. Then the medium was warmed to 37 °C and the pH adjusted to 7.4. Forty-five minutes prior to measurement cell culture medium was replaced by assay medium, followed by adding 5 mM glucose, 1 μM Oligomycin and 100 mM 2-Deoxy-D-glucose (2-DG) at different time-points. Following measurement of glycolytic activity protein concentration was determined including a standard curve. All cell lines were at least measured n = 10 times and normalized to total protein content, including a BSA standard with known protein concentrations. The data was later analysed using the Seahorse Report Generator.

### Seahorse assay for measurement of mitotic stress

Measurement of cellular respiration was performed using the Seahorse XF96e analyzer as previously described^32^. Cells were grown in either KGM Gold or RM + medium and seeded out as described above. Prior to respiration assays, cell culture medium was replaced by prewarmed, pH 7.4 adjusted assay medium supplemented with 5 mM glucose, 10 mM sodium pyruvate and 2 mM glutamate according to the manufacturer’s protocol. Oxygen consumption rate (OCR), extracellular consumption rate (ECAR) and proton production rate were measured under basal conditions and in the presence of oligomycin, a complex V inhibitor (1 μM), the complex III inhibitor antimycin A (0.5 μM), the complex I inhibitor rotenone (0.5 μM) and the mitochondrial uncoupler carbonyl-cyanide-p-trifluoromethoxyphenylhydrazone (FCCP) (1.5 μM) to assess maximal oxidative capacity. Following the respiration assay, media was removed, wells were washed once with PBS and total protein concentration was measured using the DCTM Protein Assay Kit II (BioRad). All cell lines were at least measured n = 10 times and normalized to total protein content, including a BSA standard with known protein concentrations. The data was later analysed using the Seahorse Report Generator. Spare respiratory capacity (SRC) was defined as the difference between basal and maximum respiration.

### Immunocytochemical staining

Staining was performed as described recently Cells were seeded in a 24-well plates at 2 × 10^4^ cells per well on coverslips and cultured for one day. Afterwards cells were washed once with PBS, then fixed with either 4% Formaldehyde for 15 min at RT or with ice-cold 1:1 Aceton/Methanol for 10 min at – 20 °C. In this case cells were washed two times with PBS afterwards. Following fixation, cells were permeabilized by incubating them with 0.5% Triton X-100 for 10 min. Afterwards the cells were washed twice with PBS. To block unspecific binding sites, cells were incubated for 1 h at RT with blocking solution (10% goat serum, 0.1% Tween-20 diluted in PBS) for 1 h. Primary antibodies targeting ATP5B (HPA001520, 1:100 dilution, Sigma Aldrich), FLAG (F4042, 1:2000 dilution, Sigma-Aldrich) or Cytochrome C^33^ were diluted in PBS containing 1% goat serum/0.1% Tween. After incubation over night at 4 °C, plates were washed with PBS, and fluorescently labeled secondary antibodies were added to each well diluted in PBS (1% goat serum/0.1% Tween, 1:500 dilution). Afterwards cells were washed twice with PBS, and then incubated with 1 µg/mL 4′,6-diamidino-2-phenylindole (DAPI, diluted 1:1000) for 5 min. Afterwards, coverslips were mounted onto coverslides with Immunomount (Fisher Scientific, Schwerte, Germany) for analysis. Fluorescence images were acquired on a Leica DMI 6000B microscope equipped with a Leica DFC365 FX camera and then analyzed with the Leica LAS X imaging software (v3.3.0.16799).

### Preparation of tissue microarrays

Tissue Microarray (TMA) construction was performed as previously described^34,35^. In brief, tissue cylinders with a diameter of 1.2 mm each were punched from tumor tissue blocks using a self-constructed semi-automated precision instrument and embedded in empty recipient paraffin blocks. Four μm sections were transferred to an adhesive coated slide system (Instrumedics Inc., Hackensack, NJ) for mRNA detection and immunohistochemistry. After IHC staining, 196 of 207 OPSCC tissue cylinders included in TMA blocks were suitable for evaluation after IHC staining.

### Immunohistochemical staining

Staining procedures were described previously^30,36^. Formalin-fixed, paraffin-embedded sections were deparaffinized in 100% xylene and rehydrated in decreasing concentrations of ethanol (100%, 90% and 70%). Antigen unmasking was performed by boiling tissue sections in 10 mM citric buffer for 3 min, followed by 15 min resting at RT. Blocking of unspecific antigen sites was achieved with 50% goat serum in PBS for 1 h at RT. Organotypic skin sections were incubated over night at 4 °C with antibodies targeting ATP5B (ab14730, 1:100 dilution, Abcam). Following extensive washing, sections were incubated with secondary antibodies conjugated with Alexa Fluor 488 (A-11029, Life Technologies, Carlsbad, CA, USA) for 1 h at RT. After counterstaining with DAPI, fluorescence images were acquired with a Leica DMI 6000B microscope equipped with a Leica DFC365 FX camera and analyzed with Leica LAS X imaging software (v3.3.0.16799). Immunohistochemical staining of formalin-fixed and paraffin-embedded OPSCCs were performed on tissue micro-arrays (TMA) slides using an automated Leica Bond Stainer. Lymphoid tissue served as an internal control. For TMAs, one tissue core from each tumor was punched out and transferred into a TMA recipient block. In brief, tissue cylinders with a diameter of 1.2 mm each were punched from selected tumor tissue blocks using a self-constructed semi-automated precision instrument and embedded in empty recipient paraffin blocks. Four-micrometer sections of the resulting TMA blocks were transferred to an adhesive-coated slide system (Instrumedics Inc., Hackensack, NJ, USA) for immunohistochemistry.

### Reverse transcription quantitative-PCR (RT-qPCR)

RT-qPCR was performed to quantify mRNA levels of cellular genes using a LightCycler system (Roche Diagnostics) as previously described^37^. The primers used were: ATP5B-fw 5′: GTTGGGGTTTGTGGGTCGGGTG; ATP5B-rev 5′: TTTGGCGAAGGAGATGTTTGCG; HPRT1-fw 5′: TGACACTGGCAAAACAATGCA; HPRT1-rev 5′: GGTCCTTTTCACCAGCAAGCT. ATP5B expression was normalized to HPRT1 expression levels and the control was set to 1 (n = 4 measurements).

### Patients, tumor samples and clinical data collection

Normal human skin was obtained from the Department of Dermatology of the University upon informed consent from all the subjects. Ethical approval was obtained from the ethics Committee at the University of Cologne, Germany (registration no.: 08-144). EV lesions (n = 9) were obtained from paraffin blocks removed during routine surgical excisions (for detailed information on betaHPV typing and pathology results of EV lesions, see^38^). The use of EV skin tumors for scientific studies was approved by the ethics committee of the Medical University of Warsaw, Poland. Tissue-micro-array (TMA) specimens of formalin-fixed, paraffin-embedded cervix carcinomas were obtained from the archive files of the Department of Pathology, University of Cologne. Clinical information was obtained from the patients' medical records. The collection of cervical cancer tissues was realized according to BioMaSOTA votum (approval number 13-091) at the University of Cologne, Germany. Patients diagnosed with OPSCC between 2000 and 2009 and with sufficient tumor tissue available were included in this study. Written, informed consent for medical and scientific purpose was obtained from all OPSCC patients and tumor material was used in accordance with the regional ethics committee in Giessen, Germany (AZ 95/15, dated 19th October, 2015). All methods were performed in accordance with the relevant guidelines and regulations. Patients were treated according to local guidelines at the Department of Oto-Rhino-Laryngology, Head and Neck Surgery of the University of Giessen. Available formalin-fixed, paraffin-embedded (FFPE) cancer tissue with a thickness of 2–3 mm was mandatory to produce TMA cores. Therefore, patients treated with primary chemoradiation were frequently excluded since diagnostic tumor samples were usually insufficient in size for TMA preparation. FFPE samples of 207 primary cancers of the oropharynx (C09, C10, International Classification of Diseases for Oncology (ICD-O)) were embedded in TMA blocks. Tumor staging and histological grading was assessed according to the International Union against Cancer (UICC) TNM classification (2002)^39^ and the WHO criteria for squamous cell carcinomas of the oral mucosa^40^.

### HPV-DNA genotyping and p16^INK4a^ Immunohistochemistry

HPV-status was determined retrospectively as described^41,42^. In brief, DNA was extracted from variable numbers of FFPE tissue sections depending on the tissue size (10 µm sections, approximately corresponding to 10 × 10 mm tumor tissue) using the DNeasy Blood and Tissue Kit by Qiagen, Hilden, Germany, according to manufacturer’s instructions. Extracted DNA was analyzed for mucosal high-risk HPV-DNA and HPV genotypes (16, 18, 31, 33, 35, 39, 45, 51, 52, 56, 58, 59, 68, 73, and 82) as previously described^43,44^. p16^INK4a^ expression was detected using the CINtec Histology kit (Roche mtm Laboratories, Mannheim, Germany) according to antibody suppliers’ and standard protocols^45^.

### Statistical and survival analysis

Statistical analyses were performed using SPSS statistical software (IBM SPSS 25.0, Chicago, Illinois, USA). Overall survival of OPSCC patients was calculated from initial date (date of histological diagnosis by routine biopsy) to date of death. Follow-up times of event-free patients were not censored. OS rates were calculated by the Kaplan–Meier method. Statistical significance of differences was calculated by log-rank test and chi-square test as appropriate. P-values ≤ 0.05 were considered significant for all tests^46^.

All in vitro experiments were repeated a minimum of three times. RT-qPCR data were expressed as mean ± sd. The data presented as immunoblots or images of immunohistochemical analysis are from a representative experiment, which was qualitatively similar in the replicate experiments. Statistical significance was determined with unpaired 2-tailed Student's t-test. The asterisks shown in the figures indicate significant differences between experimental groups (**p < 0.01, ***p < 0.001)^30^.

## Supplementary information

Supplementary file 1

Supplementary file 2
