# Supplementary material for: ATP synthase modulation leads to an increase of spare respiratory capacity in HPV associated cancers
Source: Sci Rep. 2020 Oct 15;10:17339. doi: 10.1038/s41598-020-74311-6 (PMC7567072; doi:10.1038/s41598-020-74311-6)
Supplement: Supplementary file 1 [file 41598_2020_74311_MOESM1_ESM.pdf]

ATP synthase modulation leads to an increase of spare respiratory capacity in HPV associated cancers

Matthias Kirschberg[1], Sandra Heuser[1], Gian Paolo Marcuzzi[1], Martin Hufbauer[1], Jens Michael Seeger[2], Anamaria Đukić[3], Vjekoslav Tomaić[3], Slawomir Majewski[4], Steffen Wagner[5], Claus Wittekindt[5], Nora Würdemann[6], Jens Peter Klussmann[6], Alexander Quaas[7], Hamid Kashkar[2], Baki Akgül[1]#

[1]Institute of Virology, University of Cologne, Medical Faculty and University Hospital Cologne, Cologne, Germany; [2]Institute for Medical Microbiology, Immunology and Hygiene (IMMIH), CECAD Research Center, University of Cologne, Cologne, Germany; [3]Division of Molecular Medicine, Ruđer Bošković Institute, Zagreb, Croatia; [4]Department of Dermatology and Venereology, Medical University of Warsaw, Warsaw, Poland; [5]Department of Otorhinolaryngology, Head and Neck Surgery, Justus-Liebig University Giessen, Germany; [6]Department of Otorhinolaryngology, Head and Neck Surgery, Medical Faculty, University of Cologne, Cologne, Germany; [7]Institute of Pathology, University Hospital Cologne, Cologne, Germany

Supplementary Figure 1

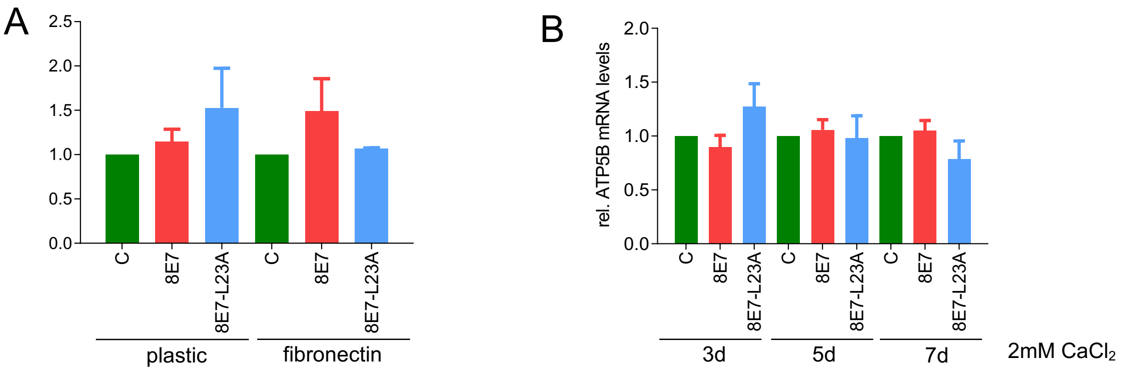

Supplementary Figure 2

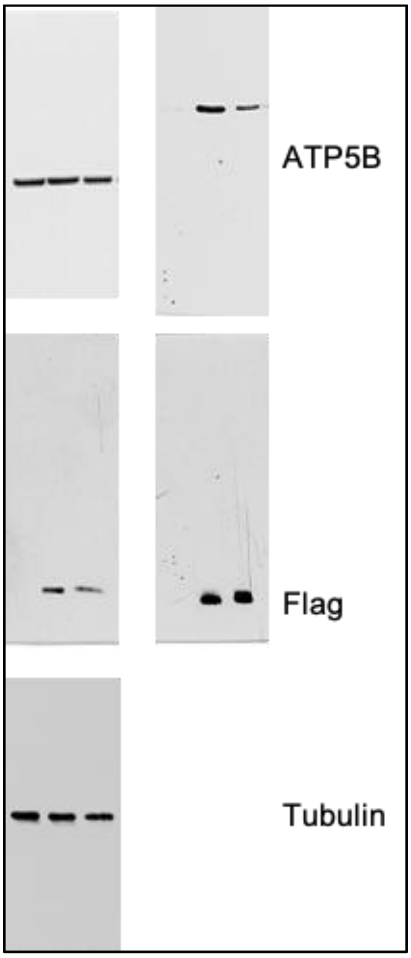

Original whole Western blots are shown. For the final figure, Photoshop was used to crop the unneeded blot areas. The whole horizontal lines of bands were adjusted. They were then placed in the Figure 1A.

Supplementary Figure 3

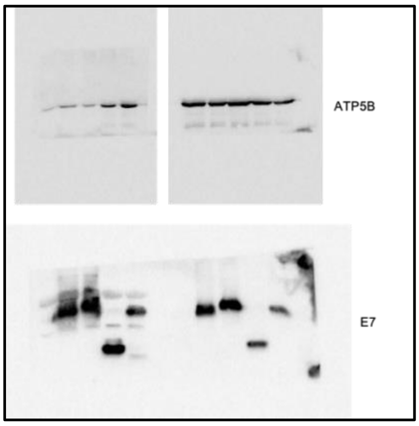

Original whole Western blots are shown. For the final figure, Photoshop was used to crop the unneeded blot areas. They were then placed in the Figure 2A.

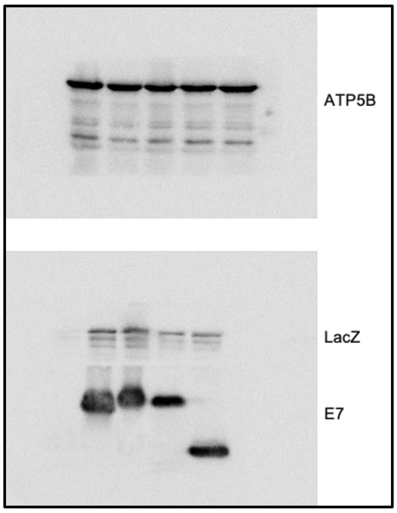

Top blot: Original whole Western blot is shown.
For the final figure, Photoshop was used to crop the lower horizontal lines of bands. Lower blot: Original whole Western blot is shown, which was cut into 2 parts before development with antibodies. For the final figure, Photoshop was used to crop the unneeded blot areas. They were then placed in the Figure 2B.

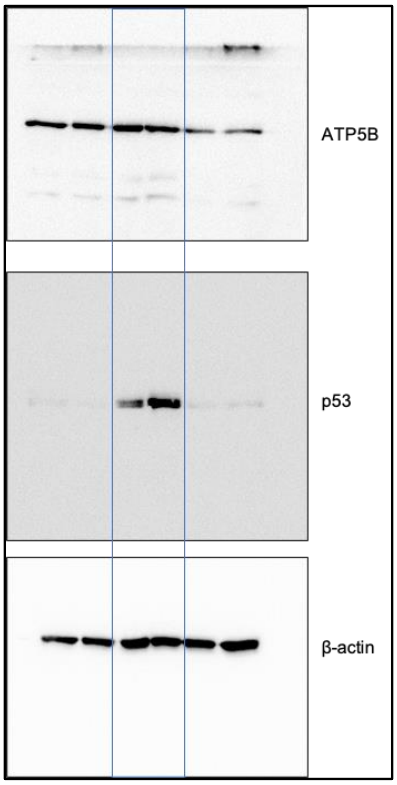

Original whole Western blot are shown. For the final figure, Photoshop was used to crop the unneeded blot areas. Then the marked bands were cropped from the image and placed together. They were then placed in the Figure 2C.

Supplementary Table 1: Ddataset for HPV8-E7 interacting cellular proteins identified by CoIP/MS in C33a cells.

P-value	LOG(P-value)	Difference	Gene names	Intensity	MS/MS Count	8E7_01	8E7_02	8E7_03	Ctrl_01	Ctrl_02	Ctrl_03
0,000007	5,1	10,9	MAP2	251230000000	271	10,5	10,4	10,9	-0,6	-0,7	0,3
0,000025	4,6	9,2	PTRF	113230000000	184	8,8	8,8	9,6	-0,3	-0,5	0,5
0,000020	4,7	9,1	HCFC1	134870000000	322	9,3	9,4	10,0	0,0	1,1	0,3
0,000090	4,0	8,9	MAP4	69491000000	120	8,5	8,4	9,0	0,5	-1,2	0,1
0,000172	3,8	8,6	CCT4	56545000000	209	7,9	8,1	8,8	-1,4	-0,1	0,5
0,000228	3,6	8,5	CCT5	55524000000	232	7,9	8,1	8,6	0,5	-1,6	0,2
0,000014	4,9	8,4	ZC3H11A	38059000000	150	7,3	7,6	8,1	-0,8	-1,0	-0,2
0,000142	3,8	8,3	CCT7	40502000000	202	7,4	7,5	8,2	0,3	-0,6	-1,5
0,000020	4,7	8,2	CCT2	53832000000	250	7,8	7,9	8,6	-0,6	0,1	0,1
0,000003	5,5	8,1	CCT8	75388000000	286	8,3	8,4	9,0	0,5	0,5	0,3
0,000020	4,7	8,1	CCT3	54139000000	206	7,8	7,8	8,6	-0,5	0,1	0,2
0,000355	3,4	8,0	TCP1	52014000000	215	7,5	7,8	8,6	-1,3	0,7	0,4
0,000122	3,9	7,9	CCT6A	42657000000	164	7,5	7,6	8,1	-0,3	0,7	-1,0
0,000065	4,2	7,8	TRIM28	34095000000	181	7,0	7,0	7,8	-0,3	0,0	-1,2
0,000010	5,0	7,7	SYNPO2	40849000000	218	7,7	7,8	8,4	0,2	0,0	0,6
0,000023	4,6	7,6	NUDC	26513000000	95	6,7	6,8	7,5	-0,1	-0,8	-0,9
0,000116	3,9	7,6	XRCC5	28549000000	176	6,8	6,8	7,6	-0,5	0,2	-1,3
0,006151	2,2	7,3	HSPA9	30040000000	169	7,4	7,4	8,0	1,2	-2,4	2,1
0,000425	3,4	6,9	EIF3A	19246000000	172	6,5	6,4	7,0	-1,3	0,8	-0,3
0,001379	2,9	6,8	RPL10;RPL10L	41278000000	138	7,7	7,5	7,3	-0,6	2,3	0,3
0,000760	3,1	6,7	DNAJA1	17534000000	64	6,1	6,3	7,1	-0,7	1,0	-1,2
0,000011	5,0	6,7	GTF2I	19468000000	149	6,5	6,6	7,3	0,1	0,1	-0,1
0,000029	4,5	6,5	LMNB1	25614000000	162	7,0	6,9	7,5	0,8	0,1	0,9
0,000025	4,6	6,5	CLTC	14675000000	138	6,0	6,1	6,7	0,0	-0,1	-0,6
0,000229	3,6	6,4	PHB2	16177000000	73	5,6	5,5	6,2	-1,5	0,0	-0,4
0,000048	4,3	6,4	COPA	10465000000	100	5,5	5,5	6,4	-0,8	-0,7	-0,3
0,000173	3,8	6,3	TUBB3	10071000000	54	5,5	5,5	6,2	0,1	-0,5	-1,3
0,003855	2,4	6,3	HSPD1	21909000000	169	6,5	6,6	7,3	0,2	2,4	-1,1
0,001267	2,9	6,2	RPL23	16580000000	84	6,0	6,4	6,9	0,1	1,5	-0,9
0,000614	3,2	6,1	HSPA1A	7890800000	80	5,0	5,0	5,7	-0,6	-0,1	-2,0
0,000261	3,6	6,1	SNRNP200	8223300000	120	5,1	5,2	5,9	-1,1	0,2	-1,3
0,000252	3,6	6,1	RBL1	10053000000	110	5,8	5,8	6,4	0,7	-0,9	-0,2
0,001208	2,9	6,1	ATP5L	4253000000	14	4,2	4,3	4,7	-2,3	-0,2	-2,3
0,000022	4,7	6,0	EPRS	8346500000	102	5,2	5,3	5,7	-0,9	-0,2	-0,7
0,000133	3,9	6,0	CACYBP	13088000000	82	5,5	5,6	6,1	-0,2	-1,0	0,3
0,001156	2,9	6,0	RPS27	16414000000	53	5,7	6,1	6,8	1,5	-0,4	-0,4
0,001499	2,8	5,9	VDAC1	15364000000	87	6,4	6,2	7,3	1,3	1,5	-0,7
0,000082	4,1	5,9	LIN54	6027400000	64	5,2	4,9	5,7	-1,0	-0,1	-0,7
0,000262	3,6	5,9	VDAC3	5835100000	43	5,0	4,9	5,7	-1,3	0,1	-0,9
0,000204	3,7	5,9	VDAC2	9978100000	51	5,8	5,7	6,5	0,1	-0,5	0,8
0,000018	4,8	5,8	CHD8	10396000000	85	5,7	5,8	6,3	0,2	0,3	-0,2
0,000030	4,5	5,8	CSE1L	9553500000	96	5,5	5,6	6,2	-0,4	0,1	0,1
0,001184	2,9	5,7	NUP93	6450100000	70	4,8	4,7	5,5	0,5	-1,7	-1,0
0,000159	3,8	5,7	AIFM1	12799000000	117	5,6	5,7	6,6	-0,2	0,4	0,7
0,000068	4,2	5,6	EIF3B	7778000000	85	4,9	4,9	5,9	-0,5	-0,4	-0,4

0,000197	3,7	5,6	RBBP4	7677300000	56	5,0	5,2	5,8	-0,6	-0,7	0,4
0,000244	3,6	5,6	CAD	9439900000	121	5,2	5,4	6,0	0,5	-0,8	0,2
0,000245	3,6	5,6	SMCHD1	5514000000	83	4,5	4,5	5,3	-1,3	-1,0	-0,1
0,000215	3,7	5,5	RPN1	8578400000	78	5,2	5,2	5,8	0,1	-0,9	0,4
0,002420	2,6	5,5	DYNC1H1	6704900000	125	4,8	4,5	5,5	0,5	-2,0	-0,2
0,002562	2,6	5,5	RPA1	6306800000	74	4,4	4,7	5,5	-1,5	0,9	-1,3
0,000049	4,3	5,5	ATAD3A	9164900000	81	5,4	5,2	6,1	-0,1	0,2	0,2
0,000094	4,0	5,4	GCN1L1	8939000000	147	5,2	5,3	6,1	0,4	0,1	-0,3
0,000329	3,5	5,4	SLAIN2	7014700000	56	5,4	5,3	6,0	1,0	-0,4	-0,1
0,004284	2,4	5,4	HSP90AA1	16864000000	115	5,9	5,9	6,6	0,6	2,3	-0,8
0,000077	4,1	5,4	DDX46	8714400000	87	5,4	5,4	5,9	0,2	-0,3	0,6
0,000379	3,4	5,3	PRKDC	9476000000	173	5,1	5,1	6,1	0,7	0,1	-0,5
0,000620	3,2	5,3	EIF3I	7370100000	58	4,7	4,9	5,8	-1,0	0,1	0,5
0,000077	4,1	5,3	CHD4	6419100000	85	4,9	4,8	5,7	0,1	-0,3	-0,3
0,000256	3,6	5,3	DNAJA2	4964600000	47	4,3	4,4	5,1	-1,3	-0,1	-0,5
0,000139	3,9	5,2	CALU	9373000000	64	5,4	5,2	6,1	0,8	0,1	0,0
0,000038	4,4	5,2	DHX15	6086600000	61	4,6	4,9	5,3	-0,3	-0,5	0,0
0,000914	3,0	5,2	GEMIN5	4874700000	70	4,5	4,3	5,4	0,5	-1,1	-0,7
0,000074	4,1	5,2	ATP5B	7978000000	71	5,3	5,4	5,8	0,1	0,0	0,9
0,000482	3,3	5,2	PRPF8	5880300000	85	4,5	4,5	5,4	0,4	-0,4	-1,0
0,000161	3,8	5,1	OGT	6095900000	73	5,2	5,2	5,7	0,1	0,9	-0,2
0,000120	3,9	5,1	EIF3G	5603500000	35	4,6	4,7	5,6	-0,1	0,1	-0,5
0,000614	3,2	5,1	SBNO1	4311500000	61	4,1	4,3	5,1	-0,7	0,2	-1,2
0,000214	3,7	5,1	TUBB6	6683400000	41	4,4	4,4	5,1	-1,1	-0,1	-0,1
0,000194	3,7	5,0	IMMT	3452600000	35	4,0	4,1	4,6	-1,0	-0,1	-1,2
0,012050	1,9	5,0	HSPA5	26151000000	175	7,0	7,0	7,7	3,0	3,7	0,0
0,000137	3,9	5,0	RAD51AP1	5184600000	30	4,6	4,7	5,4	-0,4	-0,3	0,4
0,000133	3,9	4,9	IARS	7778500000	95	4,9	4,8	5,8	0,2	0,0	0,5
0,000391	3,4	4,9	USP7	4411400000	49	3,7	3,8	4,9	-0,4	-0,7	-1,3
0,004713	2,3	4,9	YTHDF2	4791200000	51	4,5	4,3	5,0	0,5	0,5	-2,0
0,002901	2,5	4,8	XRCC6	10401000000	106	4,7	4,7	6,2	-0,3	1,5	-0,1
0,000596	3,2	4,8	ATP1A1	7792900000	102	5,1	5,2	5,9	0,3	1,4	0,0
0,000146	3,8	4,8	AAMP	4385900000	47	4,3	4,6	5,1	-0,3	-0,5	0,3
0,001064	3,0	4,8	KARS	4270000000	48	4,0	4,2	4,7	0,3	-0,3	-1,5
0,001661	2,8	4,7	PTPN14	3408200000	39	4,4	4,5	5,1	-1,2	0,8	0,2
0,000918	3,0	4,7	RPL10	6314400000	24	4,7	4,3	5,3	0,0	0,9	-0,7
0,000063	4,2	4,7	TACC2	3060400000	37	4,3	4,3	4,9	0,0	-0,5	-0,2
0,000253	3,6	4,6	TUBB8	9253200000	44	4,3	4,0	4,8	0,1	-0,8	-0,1
0,000066	4,2	4,6	CAP1	4859900000	39	4,3	4,4	5,0	-0,4	0,2	0,0
0,029977	1,5	4,5	FASN	14084000000	157	5,3	5,3	6,2	-0,4	3,8	-0,1
0,000324	3,5	4,5	SLC25A13	3206600000	45	3,9	3,6	4,8	-0,1	-0,6	-0,6
0,000389	3,4	4,5	C1QBP	4784700000	39	4,0	3,8	5,0	0,0	-0,6	-0,1
0,001039	3,0	4,5	SFXN1	4368100000	42	4,2	4,0	5,0	0,6	0,0	-0,9
0,003964	2,4	4,5	NFRKB	4547100000	42	4,4	4,7	5,4	1,2	-1,0	0,9
0,001832	2,7	4,4	SF3B1	3218600000	51	3,7	3,3	4,8	-0,2	-1,4	-0,1
0,000120	3,9	4,4	RANGAP1	2866400000	36	3,4	3,5	4,3	-0,8	-0,5	-0,8
0,001422	2,8	4,4	MAP2	7389600000	31	4,8	5,1	5,5	1,6	0,8	-0,2
0,002571	2,6	4,4	MARS	3343500000	44	3,7	3,7	4,5	0,1	0,2	-1,6
0,000398	3,4	4,4	SLC25A24	3024900000	38	3,6	3,7	4,6	-0,9	-0,3	0,0

0,000892	3,0	4,4	XPO1	3550200000	59	4,0	4,3	4,8	0,9	-0,3	-0,6
0,000844	3,1	4,4	STRAP	3728200000	46	3,7	4,0	4,5	-0,8	0,6	-0,6
0,000494	3,3	4,3	SUPT16H	5315000000	64	4,3	4,3	5,2	0,2	-0,2	0,8
0,000804	3,1	4,3	ATP2A2	5100100000	64	4,3	4,5	5,2	1,0	-0,4	0,5
0,000152	3,8	4,3	RPSA;RPSAP58	7115600000	39	4,4	4,4	5,1	0,0	0,3	0,7
0,000232	3,6	4,3	EPB41L2	3658800000	67	4,1	4,1	4,9	-0,3	0,3	0,3
0,000279	3,6	4,3	ATP5F1	2865700000	23	3,8	3,7	4,3	0,2	-0,8	-0,5
0,001224	2,9	4,3	MAGEA4	4045800000	71	4,0	4,1	4,8	0,5	-0,9	0,4
0,006183	2,2	4,3	EFTUD2	2849800000	48	3,2	3,3	4,5	0,8	-1,3	-1,2
0,011566	1,9	4,2	RANBP2	3455300000	65	3,8	4,0	4,5	1,0	0,6	-1,9
0,000389	3,4	4,2	CDK2	2179900000	26	3,7	3,7	4,1	-1,1	-0,1	0,0
0,000250	3,6	4,2	HCFC2	3146900000	41	4,0	4,1	4,8	-0,3	0,6	0,0
0,000026	4,6	4,2	CSDE1	1925300000	41	3,1	3,1	3,6	-0,9	-0,8	-1,0
0,004066	2,4	4,2	COPB2	3767200000	52	4,0	3,9	5,0	-0,3	-0,6	1,3
0,000247	3,6	4,2	ATP5A1	15052000000	103	5,6	5,9	6,6	2,1	1,6	1,9
0,000867	3,1	4,1	COPB1	3931100000	47	3,9	3,8	4,6	0,7	-0,1	-0,6
0,012344	1,9	4,1	SLC25A6	11087000000	60	5,2	5,6	6,3	-0,2	2,4	2,6
0,011983	1,9	4,0	HNRNPF	8794100000	69	4,7	5,4	5,9	0,7	3,0	0,2
0,000709	3,1	4,0	PDCD11	1690200000	34	3,0	3,1	3,6	-0,4	-1,6	-0,4
0,004172	2,4	4,0	BPTF	1974600000	37	3,6	3,3	4,7	-1,2	0,3	0,5
0,001348	2,9	4,0	CTGF	1770600000	22	2,9	3,2	3,7	0,1	-1,5	-0,8
0,007304	2,1	4,0	IPO7	1276200000	18	2,6	2,5	3,6	-0,8	-2,4	0,0
0,000387	3,4	3,9	UPF1	2152200000	33	3,0	2,9	4,0	-0,7	-0,7	-0,5
0,001445	2,8	3,9	TPX2;HCA90	2898000000	38	3,8	3,7	4,7	0,8	0,1	-0,5
0,064267	1,2	3,8	PCBP1	3251100000	29	3,8	3,6	4,8	-1,9	3,0	-0,5
0,000192	3,7	3,8	AGK	2235300000	29	2,8	3,2	3,7	-0,8	-0,3	-0,6
0,000974	3,0	3,8	EIF3C;EIF3CL	2766500000	31	3,4	3,4	4,2	-0,6	-0,4	0,6
0,000577	3,2	3,8	LARS	1963600000	29	2,8	3,0	4,0	-0,4	-0,6	-0,5
0,000474	3,3	3,8	SND1	1948800000	29	3,1	3,1	3,7	0,1	-0,9	-0,5
0,002434	2,6	3,8	SSRP1	2148500000	25	3,2	3,0	3,7	-1,2	0,5	-0,6
0,000711	3,1	3,7	AP3B1	1518200000	24	2,8	3,0	3,5	-0,5	-0,1	-1,3
0,003367	2,5	3,7	ILF2	3679500000	34	3,3	3,4	4,8	-0,6	0,5	0,5
0,000450	3,3	3,7	RAN	2609000000	25	3,5	3,5	4,3	-0,3	0,3	0,4
0,000039	4,4	3,7	PLRG1	1911200000	23	3,5	3,3	3,8	0,1	-0,4	-0,2
0,000536	3,3	3,6	AP2B1	2685700000	47	3,5	3,4	4,3	0,6	0,0	-0,3
0,001453	2,8	3,6	ESYT1	3466700000	27	3,3	3,5	4,2	0,6	-0,7	0,3
0,000930	3,0	3,6	SLC25A11	1719400000	24	2,8	3,0	3,7	-0,2	-0,1	-1,1
0,000419	3,4	3,6	SLC16A1	2760100000	19	3,5	3,7	4,6	0,3	0,5	0,2
0,005248	2,3	3,6	HSP90AB1	143180000000	305	9,0	8,9	9,7	4,4	6,2	6,2
0,006256	2,2	3,6	DDX3X;DDX3Y	5483800000	63	4,0	4,3	4,8	2,0	0,3	0,0
0,004607	2,3	3,6	CKAP5	9861600000	124	5,7	5,9	6,4	1,6	2,2	3,5
0,000450	3,3	3,6	EIF3D	1747800000	20	3,2	2,7	3,7	-0,7	-0,2	-0,2
0,000479	3,3	3,6	YBX1;YBX2;YBX3	2261200000	16	3,2	3,0	3,9	-0,4	-0,3	0,2
0,007996	2,1	3,5	KIAA1967	3917600000	63	3,7	3,9	4,8	-0,4	1,8	0,4
0,000771	3,1	3,5	CYC1	1927800000	20	2,8	2,9	3,8	-0,4	0,1	-0,7
0,001217	2,9	3,5	MATR3	25487000000	167	6,8	6,8	7,5	2,9	3,7	4,0
0,002117	2,7	3,4	TARDBP	1699800000	17	2,7	3,3	3,4	-0,2	-1,2	0,4
0,000162	3,8	3,4	SNW1	1605300000	28	3,0	3,3	3,4	0,2	-0,3	-0,5
0,000443	3,4	3,4	TOMM40	1039700000	12	2,7	2,6	3,4	-0,5	-0,8	-0,1

0,008489	2,1	3,4	GART	2476500000	45	2,7	1,4	3,6	-0,8	-1,4	-0,3
0,011244	1,9	3,4	RPL12	2158000000	21	3,0	2,8	4,4	-0,1	-0,9	1,0
0,001570	2,8	3,4	PPP6R3	1367900000	18	2,5	2,6	3,0	0,1	-0,9	-1,3
0,000285	3,5	3,4	BAG2	1370800000	14	2,6	2,8	3,4	-0,5	-0,6	-0,1
0,001216	2,9	3,4	SLC25A12	1644200000	27	3,1	2,5	3,4	-0,8	-0,6	0,2
0,005878	2,2	3,4	TECR	1867600000	17	2,8	3,2	3,7	0,8	-1,1	-0,1
0,001048	3,0	3,4	TUBB4B	371910000000	685	10,3	10,3	11,0	6,6	7,2	7,7
0,012919	1,9	3,4	RAD21	1096400000	15	2,6	2,6	3,5	-1,1	1,0	-1,2
0,003098	2,5	3,3	ZNF638	2027200000	34	3,3	3,5	3,8	0,3	-0,6	1,1
0,060869	1,2	3,3	hCG_2044799;HN	2512800000	43	3,5	3,4	3,9	-1,7	2,7	-0,2
0,003592	2,4	3,3	TOP2B	1607900000	30	2,6	2,9	3,4	0,4	-1,3	-0,1
0,003962	2,4	3,3	HADHA	2444200000	48	2,9	3,0	3,9	-0,4	0,8	-0,6
0,000998	3,0	3,3	RPL13A;RPL13a	2369500000	16	3,2	3,0	3,9	-0,1	0,6	-0,3
0,001436	2,8	3,3	PHB	2244400000	28	2,9	3,1	4,0	0,6	0,1	-0,4
0,000997	3,0	3,3	WDR43	2375400000	30	3,2	3,4	4,2	-0,1	0,5	0,6
0,004436	2,4	3,3	EML4	1441800000	20	2,5	3,2	3,4	0,7	-0,5	-0,9
0,001357	2,9	3,3	HSP90AB2P	2041000000	13	2,9	2,9	4,0	-0,2	0,3	-0,1
0,000355	3,4	3,3	TMED10	2131800000	22	3,3	3,1	3,8	0,5	-0,1	-0,1
0,010715	2,0	3,2	EMSY;C11orf30	1141900000	24	2,5	2,7	3,2	0,2	-1,8	0,3
0,000179	3,7	3,2	SLC25A3	36174000000	98	7,2	7,3	7,9	4,1	4,2	4,3
0,060074	1,2	3,2	RPS20	6192800000	45	3,7	3,9	4,3	2,4	1,5	-1,6
0,010284	2,0	3,2	KPNA2	4547400000	38	4,1	4,1	4,9	1,5	2,1	-0,1
0,002372	2,6	3,2	NUP133	1533200000	34	2,8	2,7	3,4	0,5	-0,5	-0,8
0,004901	2,3	3,2	VIM	1643800000	27	2,0	2,5	3,6	-0,5	0,1	-1,0
0,001232	2,9	3,2	AFG3L2	1142900000	25	2,3	2,2	3,1	-1,2	-0,6	-0,3
0,002073	2,7	3,2	KIF2C	2369500000	41	3,3	3,5	4,3	0,4	0,1	1,1
0,009850	2,0	3,2	RCN2	1812600000	25	2,9	2,4	3,9	0,1	0,7	-1,1
0,000512	3,3	3,1	SPCS3	1312500000	16	2,8	2,8	3,0	-0,3	0,3	-0,7
0,005103	2,3	3,1	DHX9	1751100000	25	2,6	2,1	4,0	-0,2	-0,1	-0,3
0,000818	3,1	3,1	COPG1	2342800000	52	2,8	3,2	4,0	0,3	0,0	0,3
0,001315	2,9	3,1	EIF4G1	2073500000	31	2,8	2,8	4,0	0,3	0,0	0,0
0,002572	2,6	3,1	HSD17B12	2036500000	24	2,6	3,3	3,8	-0,4	0,6	0,3
0,004395	2,4	3,1	RAD18	1237400000	18	2,4	2,9	3,4	-0,3	0,6	-0,9
0,001295	2,9	3,1	HSPA8	147330000000	373	9,2	9,2	10,0	5,9	6,4	6,9
0,003033	2,5	3,1	NACA	1885800000	20	2,9	2,7	3,3	-0,3	-0,7	0,8
0,002340	2,6	3,1	NUP107	1521700000	26	2,7	3,0	3,5	-0,6	0,7	-0,1
0,001178	2,9	3,1	TIMM50	1577000000	21	2,7	2,9	3,7	-0,1	-0,2	0,4
0,000463	3,3	3,1	TARS	1969900000	38	2,9	2,8	3,5	-0,2	0,4	-0,1
0,016266	1,8	3,0	EEF1B2	1702200000	21	2,3	2,7	3,3	-1,4	-0,5	1,0
0,003200	2,5	3,0	SLC25A5	51833000000	119	7,7	7,9	8,5	4,6	4,6	5,8
0,001941	2,7	3,0	TUBB	133440000000	184	8,7	8,5	9,8	5,9	6,0	6,1
0,003547	2,5	3,0	SMC4	1837700000	34	3,0	2,7	3,5	0,6	-0,8	0,4
0,008744	2,1	3,0	DDX21	1789200000	34	2,7	3,0	3,5	0,5	0,8	-1,1
0,034562	1,5	3,0	NDUFA4	2652400000	13	3,5	3,2	3,9	-1,2	0,9	2,0
0,003818	2,4	3,0	KIF11	950330000	17	2,4	2,4	3,2	0,2	-1,1	0,1
0,004951	2,3	3,0	MYH10	2255700000	42	2,9	2,0	3,8	0,0	-0,3	0,2
0,000935	3,0	3,0	FAF2	1177500000	18	2,5	2,3	3,4	-0,4	-0,1	-0,2
0,020889	1,7	2,9	PDS5A	2181400000	38	3,4	3,0	4,0	-0,7	0,3	1,9
0,000990	3,0	2,9	DDX42	1102200000	27	2,3	2,2	3,2	-0,3	-0,1	-0,7

0,003304	2,5	2,9	RPN2	1679400000	29	2,8	3,0	3,5	-0,6	0,8	0,4
0,011261	1,9	2,9	NUP205	1661200000	31	2,9	2,8	3,4	-1,1	0,8	0,8
0,002057	2,7	2,9	ELF2	1359500000	23	3,2	2,5	3,8	0,1	0,3	0,5
0,000084	4,1	2,9	PAF1	1274700000	22	2,8	2,5	2,8	0,0	-0,1	-0,5
0,001419	2,8	2,8	DDX20	1009500000	24	2,4	1,8	3,0	-0,6	-0,4	-0,3
0,003966	2,4	2,8	HSP90B1	1125900000	21	2,0	2,1	3,3	-0,5	-0,7	0,0
0,001595	2,8	2,8	RPL31	2862900000	17	3,2	2,7	3,4	0,2	0,8	-0,2
0,001733	2,8	2,8	YME1L1	1550000000	33	2,6	2,4	3,4	0,0	-0,5	0,3
0,007090	2,1	2,8	ERLIN1	811170000	14	1,9	2,0	2,3	0,3	-1,4	-1,2
0,010386	2,0	2,8	EIF5B	1546200000	29	2,5	2,5	3,3	0,0	-1,0	0,9
0,014265	1,8	2,8	EIF3E	1311200000	17	2,8	2,3	3,2	0,9	0,3	-1,2
0,026126	1,6	2,8	RAP1B;RAP1A	2015400000	20	2,4	3,0	3,2	1,5	0,1	-1,1
0,032718	1,5	2,7	SCCPDH	1197300000	16	1,5	1,6	3,9	0,3	-0,8	-0,7
0,006553	2,2	2,7	TUBB2B;TUBB2A	2224400000	33	2,6	2,5	3,9	-0,1	0,8	0,2
0,000465	3,3	2,7	TUBA1B;TUBA4A	319060000000	455	10,3	10,4	10,8	7,4	7,9	8,1
0,102848	1,0	2,7	RPS17L;RPS17	2665600000	26	3,4	3,2	4,0	-0,5	3,4	-0,2
0,123562	0,9	2,6	PRPF19	7409500000	54	4,8	4,7	5,5	-0,2	3,4	4,0
0,008143	2,1	2,6	RARS	989830000	28	2,0	2,4	3,0	0,3	-1,0	0,2
0,002741	2,6	2,6	SMARCA5	1534400000	28	2,6	2,6	3,6	-0,1	0,6	0,6
0,065272	1,2	2,6	PGAM5	2361200000	25	2,8	3,5	4,1	-0,4	0,2	2,8
0,000899	3,0	2,6	SMC2	936830000	26	2,2	2,2	2,7	0,2	-0,5	-0,4
0,002751	2,6	2,6	OXA1L	1691300000	19	2,6	2,8	2,8	0,6	0,6	-0,6
0,033594	1,5	2,5	ACLY	1124700000	27	1,8	2,0	2,8	0,9	-0,2	-1,6
0,015112	1,8	2,5	ARCN1	1291300000	21	2,2	1,9	3,2	-0,4	-0,6	0,9
0,018109	1,7	2,5	CDC73	1182400000	26	2,3	2,3	3,4	0,2	1,1	-0,7
0,004963	2,3	2,4	TRIP13	857600000	13	2,1	2,0	2,8	-0,1	-0,8	0,4
0,087406	1,1	2,4	ERLIN2	2895600000	25	3,8	3,5	4,0	0,4	0,2	3,5
0,025797	1,6	2,4	NCAPH	1159400000	18	2,5	2,1	2,8	-1,3	0,6	0,9
0,003622	2,4	2,4	NDUFS1	631860000	20	1,0	1,9	2,1	-0,4	-1,1	-0,7
0,003244	2,5	2,4	NOMO1;NOMO3;I	792850000	19	1,9	2,1	2,5	-0,9	-0,1	0,2
0,051283	1,3	2,4	HNRNPD	2416400000	20	3,4	2,9	4,2	2,5	1,0	-0,2
0,006498	2,2	2,3	ATP5C1	1300500000	18	2,5	2,5	3,1	0,0	1,1	-0,2
0,015532	1,8	2,3	SF3A1	821570000	15	2,2	1,9	2,5	0,6	0,3	-1,2
0,016576	1,8	2,3	DARS	935830000	15	2,0	1,7	3,0	-0,9	0,3	0,3
0,026855	1,6	2,3	NOP58	1005400000	17	1,8	2,4	2,5	0,7	-1,3	0,5
0,004848	2,3	2,3	EEF1A1P5;EEF1A1	51669000000	262	7,4	7,5	8,3	5,4	6,0	4,9
0,011440	1,9	2,3	NOC2L	1542900000	20	2,6	2,1	3,7	0,6	0,1	0,8
0,009659	2,0	2,3	SLC3A2	1192100000	21	1,8	2,2	3,2	0,5	-0,3	0,3
0,000124	3,9	2,3	C2orf47	715870000	15	1,8	1,9	2,2	-0,1	-0,3	-0,4
0,025249	1,6	2,2	CLINT1	691540000	13	2,0	1,7	2,4	-0,6	-1,0	1,0
0,041262	1,4	2,2	EEF1G	5962800000	38	4,5	4,6	5,0	1,0	3,2	3,3
0,071715	1,1	2,2	AP1M1	1164900000	16	1,9	1,8	2,8	0,8	-1,7	0,8
0,001401	2,9	2,2	DLAT	680480000	20	1,4	1,6	2,3	-0,5	-0,5	-0,3
0,024959	1,6	2,2	G3BP1	684790000	19	1,5	1,5	2,2	-1,2	-0,8	0,7
0,017916	1,7	2,2	COPG2	1551800000	29	2,5	2,6	3,2	0,1	1,6	0,0
0,008566	2,1	2,2	AP3D1	410020000	15	1,1	0,8	1,4	-0,2	-1,2	-1,7
0,050827	1,3	2,2	ZNF687	657660000	14	2,1	2,3	0,0	-1,1	-0,7	-0,3
0,006101	2,2	2,1	POLR2B	552140000	11	1,7	1,5	2,1	-0,2	0,1	-1,1
0,003320	2,5	2,1	RPLP0;RPLP0P6	39649000000	169	7,2	7,2	8,0	5,0	5,4	5,6

0,000733	3,1	2,1	RPS3	68663000000	220	7,9	8,1	8,6	6,1	6,2	6,1
0,030621	1,5	2,1	LARP4	337680000	9	0,9	0,1	2,1	-1,3	-0,6	-1,3
0,008960	2,0	2,1	TMCO1	785550000	12	1,6	1,8	2,4	-0,5	0,6	-0,6
0,020699	1,7	2,0	TMEM33	1367500000	16	1,8	2,0	3,3	0,1	1,0	0,0
0,065266	1,2	2,0	FARSB	592050000	16	0,5	1,1	2,7	-0,2	-1,4	0,0
0,041682	1,4	2,0	NDUFA8	745390000	14	1,3	0,5	2,7	-0,3	-0,3	-0,9
0,030540	1,5	2,0	EEF2	1212000000	39	2,2	2,0	3,0	0,8	-0,6	1,0
0,018159	1,7	2,0	TFRC	764150000	16	1,0	1,5	2,7	0,0	-0,5	-0,2
0,014395	1,8	2,0	VARS	1557400000	24	2,4	2,5	3,4	1,0	0,1	1,3
0,042119	1,4	1,9	SYPL1	529200000	11	1,6	1,6	2,2	-0,3	1,0	-1,2
0,018932	1,7	1,9	ZMYM6	418440000	7	1,4	1,5	2,3	0,4	-0,1	-1,0
0,053000	1,3	1,9	THOC2	834040000	20	1,8	0,2	2,4	-0,2	-0,4	-0,7
0,010772	2,0	1,8	RPS27A;UBB;UBC;	28917000000	68	7,0	6,8	7,7	4,9	5,2	5,9
0,020391	1,7	1,8	CYFIP1	815250000	17	2,1	1,8	2,8	0,0	0,2	1,2
0,030643	1,5	1,8	CCNA2	410700000	15	1,7	1,7	2,3	-0,7	1,0	0,2
0,032754	1,5	1,7	USP10	393680000	14	0,6	1,8	2,3	-0,3	0,1	-0,2
0,006963	2,2	1,7	HNRNPU	43735000000	191	7,2	7,2	8,2	5,8	6,0	5,8
0,015841	1,8	1,6	WAPAL	75397000	2	1,1	0,7	0,7	-0,7	-0,1	-1,4
0,001563	2,8	-1,4	CSTB	100860000	3	-0,8	-0,9	-0,3	0,7	1,0	0,7
0,007197	2,1	-1,5	IGKV2D-29	1528000000000	446	11,8	11,7	12,4	13,1	13,5	13,8
0,008688	2,1	-1,5	DSC1	4355800000	50	3,8	4,3	4,2	5,5	5,2	6,1
0,017706	1,8	-1,5	SEC24A	5145200	1	-0,4	-1,1	-1,3	0,5	0,1	1,1
0,009085	2,0	-1,6	LAMB3	31961000	2	-0,7	-1,0	-0,9	1,2	0,7	0,1
0,016016	1,8	-1,6	EIF4A3	232030000	6	-1,1	-1,1	-1,7	0,3	1,0	-0,3
0,027487	1,6	-1,6	LETM1	318030000	3	-1,1	-0,4	-2,1	0,4	0,5	0,5
0,011576	1,9	-1,7	CPD	27984000	2	-2,0	-2,0	-1,4	0,5	-0,6	-0,3
0,004601	2,3	-1,7	CSTA	5059200000	45	4,2	4,0	4,3	6,2	5,3	6,1
0,040002	1,4	-1,7	CHAMP1	62556000	2	-1,6	-1,6	-0,9	-0,7	1,0	0,7
0,004637	2,3	-1,8	MYO1C	4242800000	101	3,5	3,6	4,2	5,2	5,5	6,0
0,007544	2,1	-1,8		428560000000	68	9,7	10,4	10,3	11,3	12,1	12,3
0,009910	2,0	-1,8	YWHAG;YWHAH	271600000	3	-1,4	-1,2	-0,6	0,2	0,9	1,2
0,043555	1,4	-1,8	HTATSF1	230100000	4	-1,8	-0,3	-2,1	1,0	0,0	0,3
0,005440	2,3	-1,9	DSG1	5540400000	73	4,0	4,7	4,3	6,3	5,7	6,7
0,004710	2,3	-2,1	PRMT5	84238000000	323	7,5	7,6	8,5	9,6	9,9	10,4
0,081112	1,1	-2,1	GSDMA	742000000	15	1,9	2,3	-0,5	3,3	2,8	3,8
0,083575	1,1	-2,1	CAPZB	822910000	11	-0,4	0,1	-1,3	0,6	0,9	3,2
0,063356	1,2	-2,2	SERPINB12	635750000	17	1,6	1,8	-0,7	3,1	2,6	3,6
0,005232	2,3	-2,3	WDR77	29628000000	133	6,0	6,0	6,9	8,2	8,5	9,1
0,001663	2,8	-2,3	ERH	32092000000	77	6,1	6,5	6,8	8,3	8,8	9,1
0,091755	1,0	-2,3	SHROOM3	666850000	5	-0,4	-0,3	-0,6	0,9	0,8	4,0
0,029566	1,5	-2,4	RBM10	240030000	5	-0,9	-0,9	-0,7	1,5	0,3	2,8
0,067602	1,2	-2,5		652060000	7	-1,1	-0,8	-0,2	1,3	0,4	3,7
0,022466	1,6	-2,6	ACTC1;ACTG2;AC	56744000000	113	6,6	5,4	6,8	7,9	9,9	8,7
0,126432	0,9	-2,7	CAT	438260000	12	-0,2	0,2	-0,6	3,7	-0,3	3,9
0,071614	1,1	-2,8	RBMX	1860100000	35	-0,5	2,5	3,2	4,2	4,5	4,8
0,069343	1,2	-2,8	STK38	1096400000	24	2,2	2,4	-1,1	3,7	3,9	4,3
0,001511	2,8	-3,1	THRAP3	16261000000	125	4,6	5,1	5,8	7,9	8,4	8,5
0,053628	1,3	-3,1	DSP	3581000000	102	3,3	4,2	0,5	6,0	5,1	6,4
0,055850	1,3	-3,2	ARPC3	355560000	6	-1,0	-1,2	-1,2	3,1	3,3	-0,4

0,010452	2,0	-3,2	ARPC5	173700000	6	-1,2	-1,5	-1,2	0,5	2,3	2,9
0,000085	4,1	-3,2	TPP2	170940000	10	-1,0	-1,2	-0,9	1,8	2,3	2,4
0,093380	1,0	-3,3	TXN	1299800000	17	0,0	-0,5	4,1	4,3	4,0	5,2
0,114690	0,9	-3,3	AZGP1	423010000	7	-0,1	-2,2	-1,0	3,6	-0,9	3,9
0,001024	3,0	-3,4	CFL1	583610000	15	-1,1	-1,0	0,0	2,3	2,6	3,0
0,000528	3,3	-3,4	ACTG1	707830000000	772	9,8	9,9	10,7	13,2	13,5	13,8
0,001432	2,8	-3,4	BCLAF1	10133000000	90	3,6	4,0	4,9	7,1	7,8	7,8
0,000514	3,3	-3,5	CALM1;CALM2;CA	2845000000	19	1,9	2,6	2,9	5,7	6,0	6,3
0,001072	3,0	-3,5	POLDIP3	478710000	10	-0,8	0,4	0,0	2,9	3,7	3,6
0,000469	3,3	-3,6	CAPZA1	9092600000	84	3,8	3,5	4,3	7,0	7,6	7,7
0,002516	2,6	-3,7	RIOK1	577670000	13	-0,6	1,0	0,1	3,4	3,7	4,4
0,000146	3,8	-3,7	ACTR2	394250000	9	-0,5	-0,4	0,1	3,2	3,3	3,8
0,000446	3,4	-3,7	CAPZB	13510000000	93	4,0	4,2	4,8	7,6	8,0	8,4
0,000145	3,8	-3,7	ACTR3	580520000	16	0,0	0,2	0,5	3,8	3,8	4,4
0,034937	1,5	-3,8	CASP14	1080300000	16	2,8	-1,0	-0,3	4,4	3,8	4,6
0,000171	3,8	-3,9	ACTN4	415460000	14	-1,1	-1,5	-0,6	2,7	2,8	3,0
0,001420	2,8	-4,2	KPRP	453050000	13	-0,4	-0,9	-1,8	3,1	2,6	3,8
0,000277	3,6	-4,8	ARPC2	482810000	12	-1,8	-0,8	-0,7	3,4	3,6	4,1
0,000031	4,5	-5,7	CLNS1A	1482900000	10	-0,6	-0,3	-0,7	5,2	4,8	5,6
0,000159	3,8	-5,8	CAPZA2	2264800000	34	0,2	-0,9	0,5	5,6	5,7	6,0
0,014289	1,8	-6,2	TMOD3	6812600000	56	0,4	-1,4	3,6	6,7	6,8	7,7
0,000323	3,5	-6,3	LIMA1	1920800000	35	-0,1	-0,2	-1,7	5,4	5,5	6,1
0,001020	3,0	-6,9	DBN1	13128000000	67	0,7	0,6	3,0	8,0	8,4	8,7
0,000495	3,3	-7,2	CTTN	4785800000	48	1,0	-1,0	-0,8	6,5	7,0	7,4
0,000003	5,5	-7,8	ACTB	11791000000	27	-0,2	-0,9	-0,6	7,1	7,4	7,2

Supplementray Table 2: HPV8-E7 interaction partners identified in yeast-two-hybrid experiments

protein ID	gene name	subcellular localization
ARFIP1	ADP-Ribosylation Factor Interacting Protein 1	Mitochondria
ATP5B	ATP synthese, H+ transporting, F1-complex, beta-polypeptide	Mitochondria
COX2	Cytochrome c oxidase II	Mitochondria
COX3	Cytochrome c oxidase III	Mitochondria
GHITM/Derp2	Growth hormone inducible transmembrane protein	Mitochondria
PARP	Poly (ADP-Ribose) Polymerase 1	Mitochondria
BLM	Bloom Syndrome, RecQ Helicase-Like	Nucleus
NCOA4	Nuclear receptor coactivator 4	Nucleus
PTRF	Polymerase I and transkript release factor	Nucleus, cell membrane
ALMS1	Alstrom Syndrome Protein 1	Cytoplasm
AP1B1	Adaptor-Related Protein Complex 1, Beta 1 Subunit	Cytoplasm
RPL26	Ribosomal Protein L26	Cytoplasm

Supplementray Table 3: Clinicopathological features of the patient cohort with OPSCC (N = 207)

		All		HPV-related		HPV-negative		
**Risk factors**		(n = 207)	100%	(n = 39)	19%	(n = 167)	81%	p[a]
Nicotine	never	26	*13%*	10	*28%*	15	*9%*	**0.002***
	former / current	177	*87%*	26	*72%*	151	*91%*	
Alcohol	≤ 2 drinks / day	65	*42%*	28	*93%*	37	*30%*	**<0.001***
	> 2 drinks / day	91	*58%*	2	*7%*	88	*70%*	
Age	young (< 60 years)	111	*54%*	21	*54%*	89	*53%*	0.950
	old (≥ 60 years)	96	*46%*	18	*46%*	78	*47%*	
Gender	male	168	*81%*	31	*79%*	137	*82%*	0.712
	female	39	*19%*	8	*21%*	30	*18%*	
ECOG	healthy (0-1)	143	*69%*	29	*74%*	113	*68%*	0.444
	sick (2-4)	63	*31%*	10	*26%*	53	*32%*	
**Tumor characteristics**								
Localization	tonsil	140	*74%*	21	*58%*	27	*18%*	**<0.001***
	other than tonsil	49	*26%*	15	*42%*	125	*65%*	
Grading	low (G1-2)	83	*49%*	15	*44%*	68	*50%*	0.515
	high (G3-4)	86	*51%*	19	*56%*	67	*50%*	
UICC stages	I - III	67	*33%*	14	*37%*	53	*32%*	0.577
	> III	136	*67%*	24	*63%*	112	*68%*	
T-stage	T1-3	161	*79%*	33	*89%*	127	*77%*	0.098
	T> 3	42	*21%*	4	*11%*	38	*23%*	
N-stage	N0	51	*55%*	6	*16%*	45	*27%*	0.136
	N > 0	152	*75%*	32	*84%*	119	*73%*	
M-stage	M0	188	*94%*	36	*97%*	151	*94%*	0.401
	M > 0	11	*6%*	1	*3%*	10	*6%*	
Recurrence	no	174	*84%*	37	*95%*	137	*82%*	**0.046**
	yes	33	*16%*	2	*5%*	30	*18%*	
