# Supplementary material for: ATP synthase modulation leads to an increase of spare respiratory capacity in HPV associated cancers
Source: Sci Rep. 2020 Oct 15;10:17339. doi: 10.1038/s41598-020-74311-6 (PMC7567072; doi:10.1038/s41598-020-74311-6)
Supplement: Supplementary file 2 [file 41598_2020_74311_MOESM2_ESM.docx]

Supplementary Figure 1: Effect of fibronectin and extracellular Calcium on ATP5B expression. (A) Relative ATP5B mRNA expression measured by RT-qPCR in N/TERT keratinocytes either expressing the control vector pLXSN, pLXSN-HPV8-E7 or pLXSN-HPV8-E7L23A grown on either plastic or fibronectin. (B) Relative ATP5B mRNA expression in N/TERT keratinocytes grown on plastic for 24, 48 and 72 hours after addition of 2mM CaCl_2_ to induce differentiation.

Supplementary Figures 2 and 3: Original Western blots shown in Fig. 1A and 2.

Supplementary Table 1: Dataset for HPV8-E7 interacting cellular proteins identified by CoIP/MS in C33a cells.

Supplementary Table 2: HPV8-E7 interaction partners identified in yeast-two-hybrid experiments

Supplementary Table 3: Clinicopathological features of the patient cohort with OPSCC (n= 207). ^a^p-values calculated by x² test (Pearson, asymptotic, two-sided), significant p-values (p≤ 0.05) in bold, *: values remaining significant after adjusting the level of significance according to Bonferroni´s correction for multiple comparisons (p≤ 0.0042 for n=12 tests).
